# The genome sequence of the Green Pug moth, *Pasiphila rectangulata *(Linnaeus, 1758)

**DOI:** 10.12688/wellcomeopenres.21224.1

**Published:** 2024-05-15

**Authors:** Denise C. Wawman, University of Oxford and Wytham Woods Genome Acquisition Lab, Darwin Tree of Life Barcoding collective, Wellcome Sanger Institute Tree of Life Management, Samples and Laboratory team, Wellcome Sanger Institute Scientific Operations: Sequencing Operations, Wellcome Sanger Institute Tree of Life Core Informatics team, Tree of Life Core Informatics collective, Darwin Tree of Life Consortium

**Affiliations:** 1 Edward Grey Institute, Department of Biology, University of Oxford, Oxford, England, UK

**Keywords:** Pasiphila rectangulata, Green Pug, genome sequence, chromosomal, Lepidoptera

## Abstract

We present a genome assembly from an individual male *Pasiphila rectangulata* (the Green Pug; Arthropoda; Insecta; Lepidoptera; Geometridae). The genome sequence is 582.5 megabases in span. Most of the assembly is scaffolded into 30 chromosomal pseudomolecules, including the Z sex chromosome. The mitochondrial genome has also been assembled and is 15.74 kilobases in length. Gene annotation of this assembly on Ensembl identified 17,153 protein coding genes.

## Species taxonomy

Eukaryota; Opisthokonta; Metazoa; Eumetazoa; Bilateria; Protostomia; Ecdysozoa; Panarthropoda; Arthropoda; Mandibulata; Pancrustacea; Hexapoda; Insecta; Dicondylia; Pterygota; Neoptera; Endopterygota; Amphiesmenoptera; Lepidoptera; Glossata; Neolepidoptera; Heteroneura; Ditrysia; Obtectomera; Geometroidea; Geometridae; Larentiinae; *Pasiphila*; *Pasiphila rectangulata* (Linnaeus, 1758) (NCBI:txid572874).

## Background

The Green Pug *Pasiphila rectangulata* is one of a group of small moths in the family Geometridae that are commonly known as pugs, apparently due to their resemblance to the flat-nosed dogs ( Marren, 2019). When freshly emerged, the main form is bright green and has a blackish belt around the abdomen, however, in some urban areas, such as London, the English Midlands and northern England, a uniformly dark brown form, f. *anthrax*, predominates ( Skinner & Wilson, 2009; Waring *et al.*, 2017).

*Pasiphila rectangulata* is a native species in the Palearctic Region, but has been accidentally introduced to North America, where it was first detected in 1970, and possibly to Japan where it was first described in 1957 ( Maier, 2005). Eggs overwinter in cracks on trees and the distinctive greenish larvae, with a mid-dorsal stripe, emerge to feed on the buds, flowers and leaves of a range of trees and shrubs, including apples and crab-apples *Malus* spp., pear *Pyrus* spp. cherries *Prunus* spp., hawthorn *Crataegus monogyna*, blackthorn *Prunus spinosa*, and common Juneberry *Amelanchier canadensis* ( Maier, 2005; Skinner & Wilson, 2009; Waring *et al*., 2017). The larvae can cause significant damage in orchards ( Maier, 2005). *P. rectangulata* is single brooded, with the adults flying from June until July or August, and can be attracted to light ( Skinner & Wilson, 2009; Waring *et al.*, 2017).

*Pasiphila rectangulata* has previously been partially sequenced – one mitochondrial and seven nuclear markers ( Lee *et al.*, 2018) – and RNA sequencing has been used to detect novel viruses in the families Nyamiviridae and Bunyaviridae from specimens caught in a suburban area of Seattle, Washington ( Makhsous *et al.*, 2017).

We present a chromosomally complete genome sequence for *Pasiphila rectangulata*, based on one specimen of the green morphotype collected using a mercury vapour light trap in a rural garden in the hamlet of Bratton, near Minehead, in Somerset, as part of the Darwin Tree of Life Project.

## Genome sequence report

**Figure 1.**
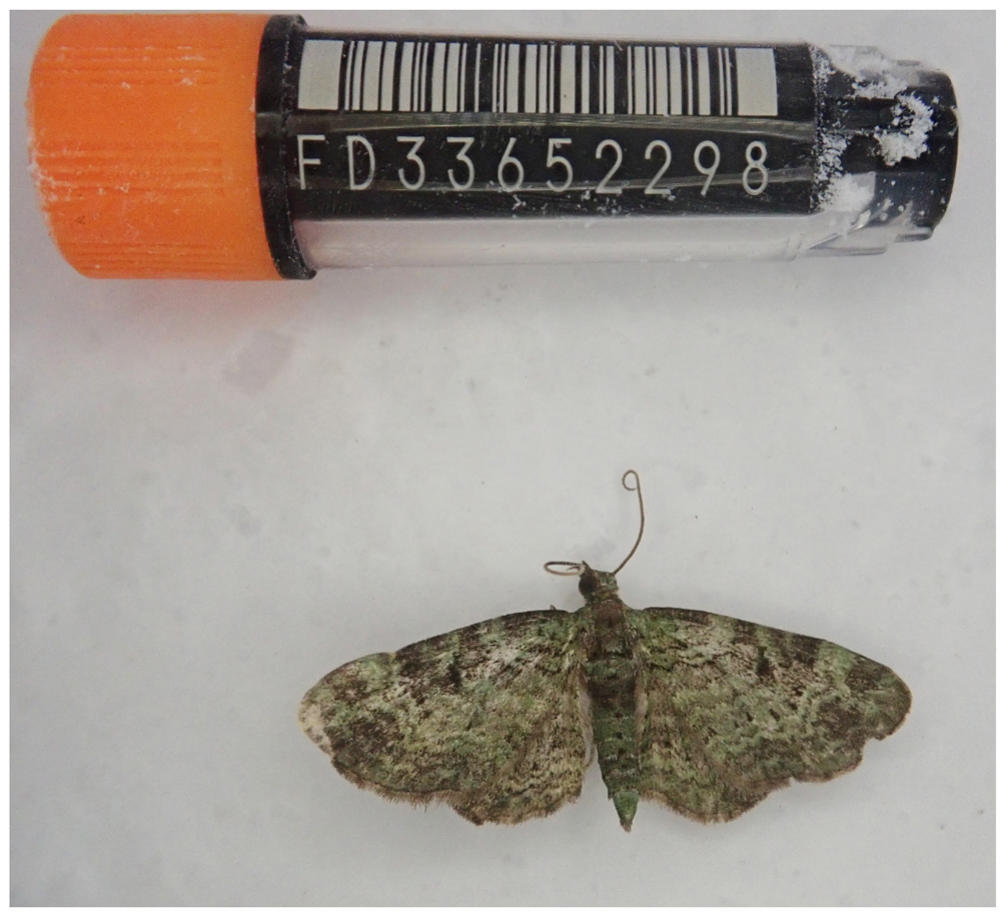
Photograph of the *Pasiphila rectangulata* (ilPasRect1) specimen used for genome sequencing.

The genome was sequenced from one male *Pasiphila rectangulata* ( Figure 1) collected from Bratton, Somerset, UK (51.20, –3.51). A total of 43-fold coverage in Pacific Biosciences single-molecule HiFi long reads was generated. Primary assembly contigs were scaffolded with chromosome conformation Hi-C data. Manual assembly curation corrected 12 missing joins or mis-joins and removed 3 haplotypic duplications, reducing the scaffold number by 3.70%.

**Figure 2.**
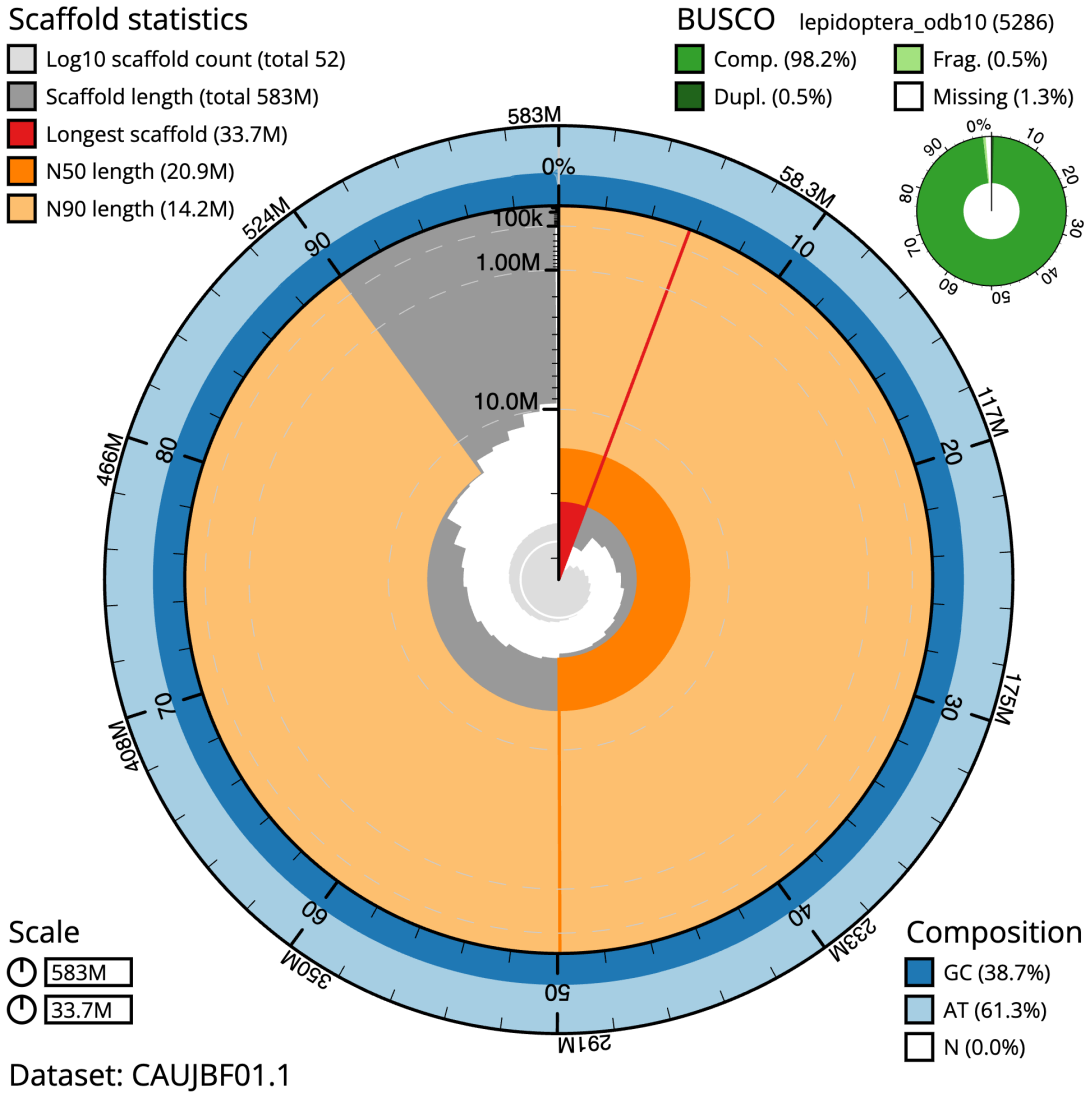
Genome assembly of *Pasiphila rectangulata*, ilPasRect1.1: metrics. The BlobToolKit snail plot shows N50 metrics and BUSCO gene completeness. The main plot is divided into 1,000 size-ordered bins around the circumference with each bin representing 0.1% of the 582,513,096 bp assembly. The distribution of scaffold lengths is shown in dark grey with the plot radius scaled to the longest scaffold present in the assembly (33,741,088 bp, shown in red). Orange and pale-orange arcs show the N50 and N90 scaffold lengths (20,901,828 and 14,179,607 bp), respectively. The pale grey spiral shows the cumulative scaffold count on a log scale with white scale lines showing successive orders of magnitude. The blue and pale-blue area around the outside of the plot shows the distribution of GC, AT and N percentages in the same bins as the inner plot. A summary of complete, fragmented, duplicated and missing BUSCO genes in the lepidoptera_odb10 set is shown in the top right. An interactive version of this figure is available at https://blobtoolkit.genomehubs.org/view/CAUJBF01.1/dataset/CAUJBF01.1/snail.

**Figure 3.**
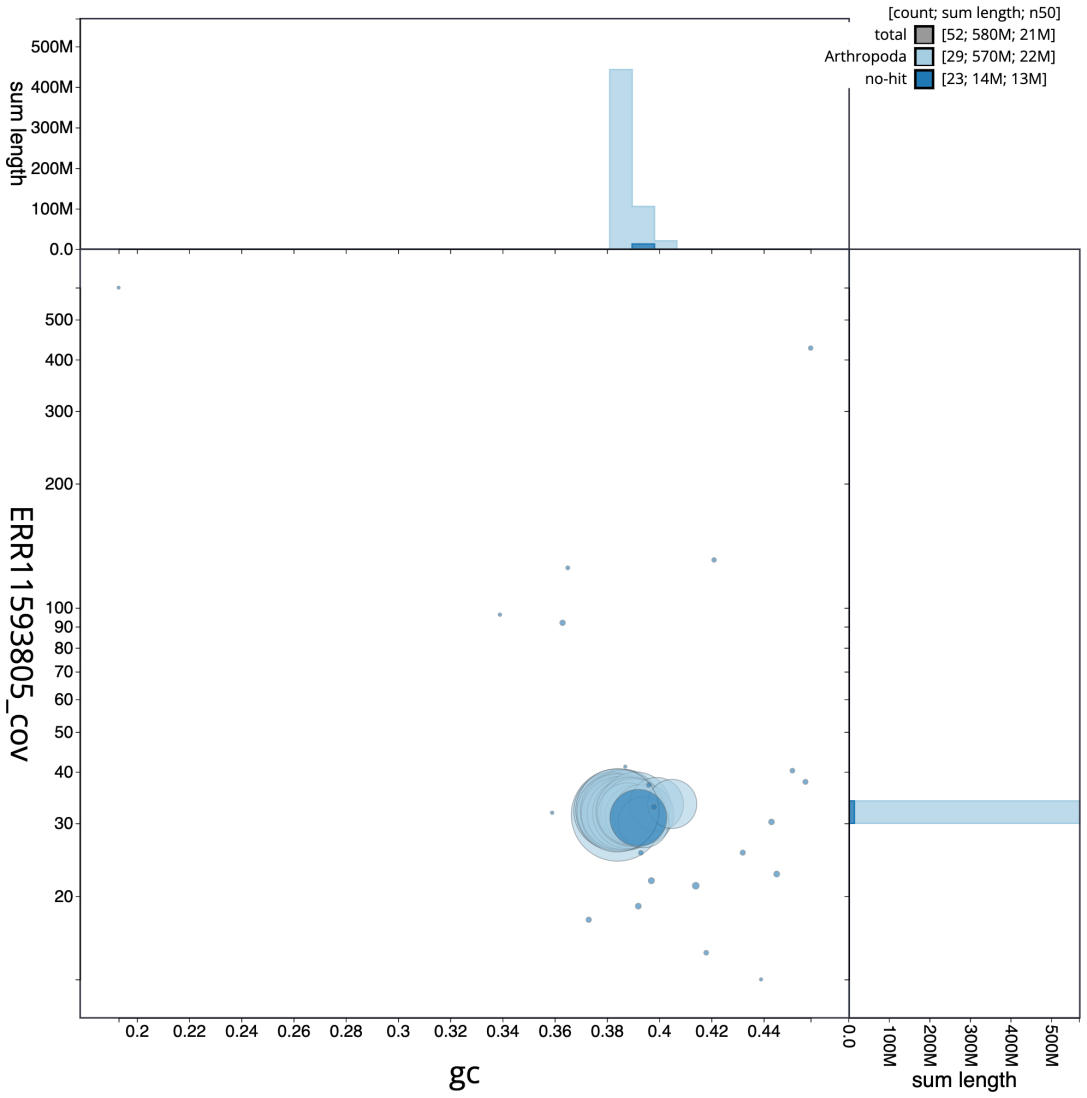
Genome assembly of *Pasiphila rectangulata*, ilPasRect1.1: BlobToolKit GC-coverage plot. Sequences are coloured by phylum. Circles are sized in proportion to sequence length. Histograms show the distribution of sequence length sum along each axis. An interactive version of this figure is available at https://blobtoolkit.genomehubs.org/view/CAUJBF01.1/dataset/CAUJBF01.1/blob.

**Figure 4.**
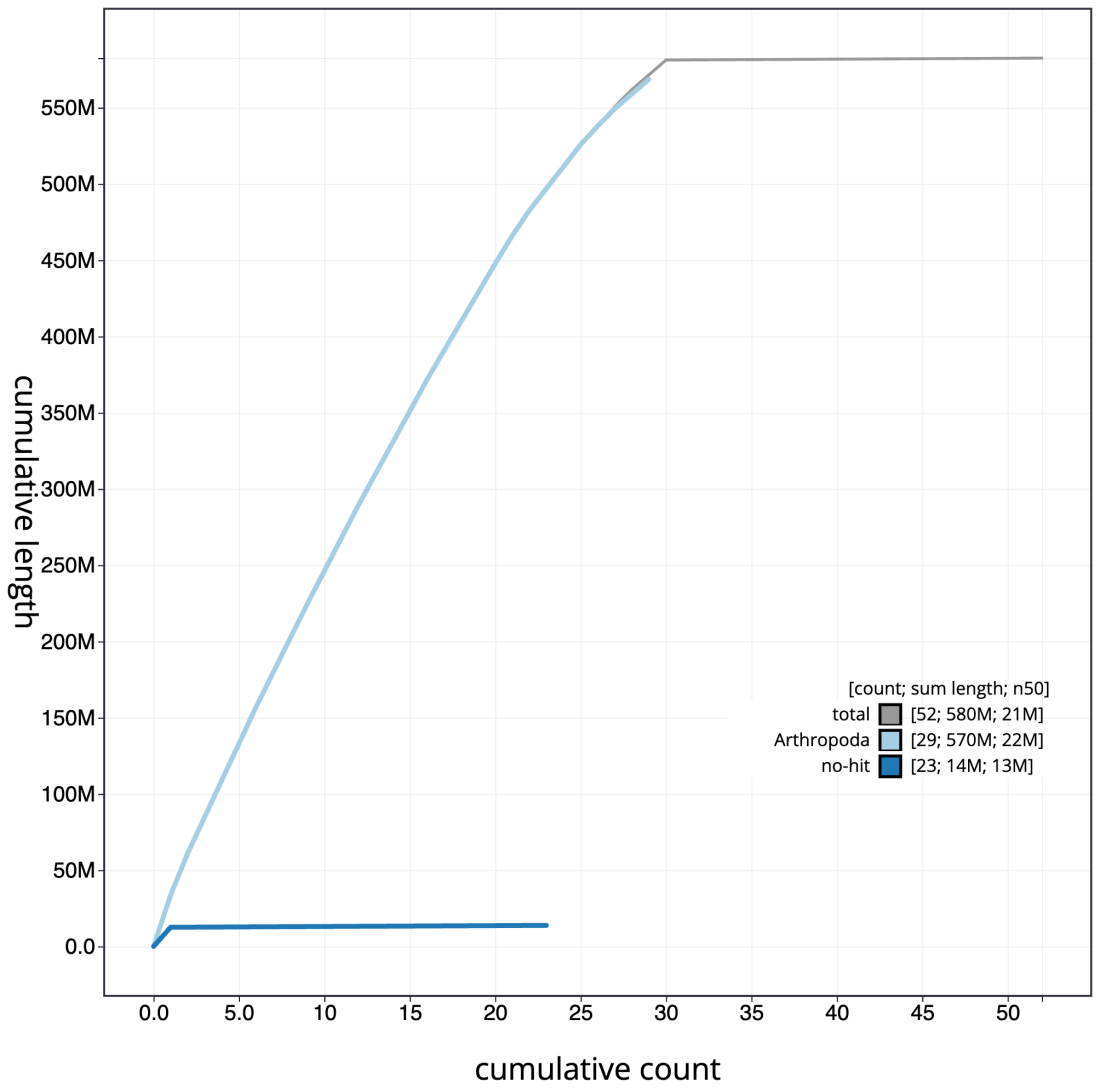
Genome assembly of *Pasiphila rectangulata*, ilPasRect1.1: BlobToolKit cumulative sequence plot. The grey line shows cumulative length for all sequences. Coloured lines show cumulative lengths of sequences assigned to each phylum using the buscogenes taxrule. An interactive version of this figure is available at https://blobtoolkit.genomehubs.org/view/CAUJBF01.1/dataset/CAUJBF01.1/cumulative.

**Figure 5.**
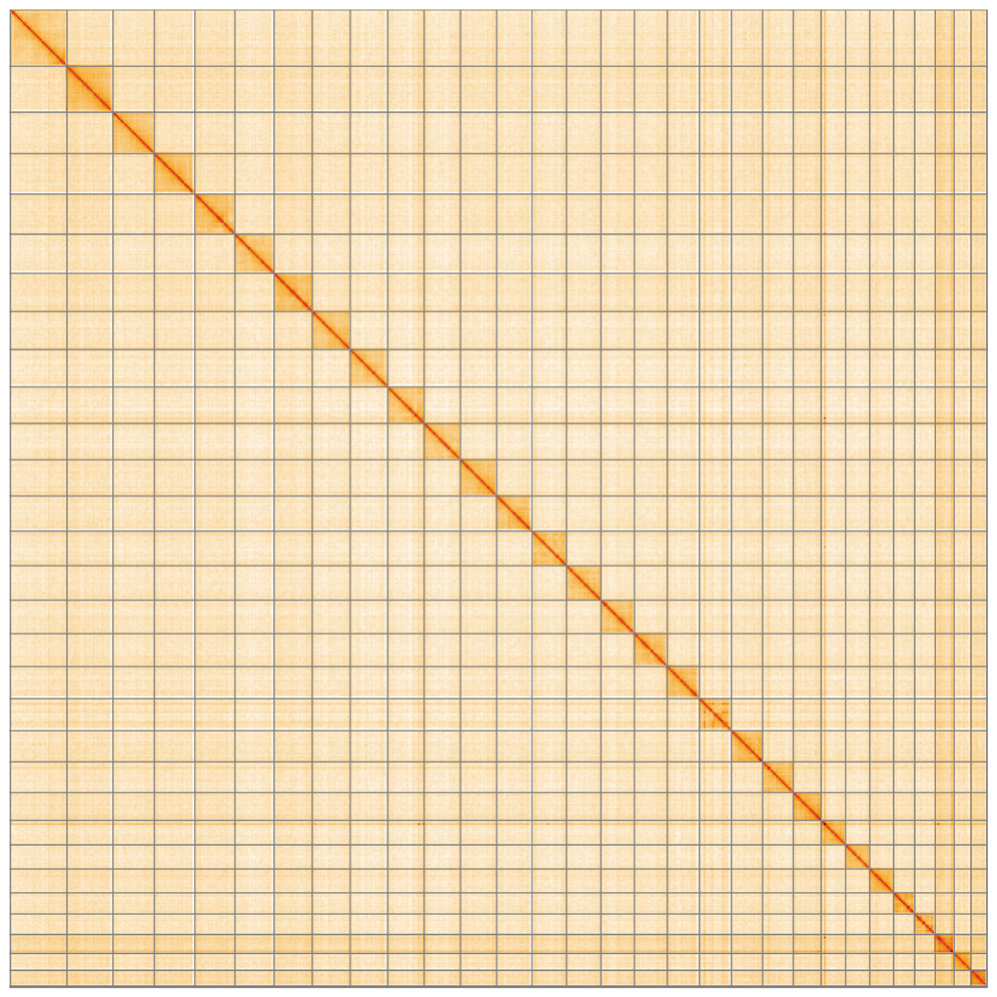
Genome assembly of *Pasiphila rectangulata*, ilPasRect1.1: Hi-C contact map of the ilPasRect1.1 assembly, visualised using HiGlass. Chromosomes are shown in order of size from left to right and top to bottom. An interactive version of this figure may be viewed at https://genome-note-higlass.tol.sanger.ac.uk/l/?d=P5lT0Q1CS3aPR2gv7Qh76A.

The final assembly has a total length of 582.5 Mb in 51 sequence scaffolds with a scaffold N50 of 20.9 Mb ( Table 1). The snail plot in Figure 2 provides a summary of the assembly statistics, while the distribution of assembly scaffolds on GC proportion and coverage is shown in Figure 3. The cumulative assembly plot in Figure 4 shows curves for subsets of scaffolds assigned to different phyla. Most (99.78%) of the assembly sequence was assigned to 30 chromosomal-level scaffolds, representing 29 autosomes and the Z sex chromosome. Chromosome-scale scaffolds confirmed by the Hi-C data are named in order of size ( Figure 5; Table 2). Chromosome Z was assigned based on synteny with *Perizoma affinitatum* (GCA_961405105.1) and *Camptogramma bilineatum* (GCA_958496255.1). While not fully phased, the assembly deposited is of one haplotype. Contigs corresponding to the second haplotype have also been deposited. The mitochondrial genome was also assembled and can be found as a contig within the multifasta file of the genome submission.

**Table 1.** Genome data for *Pasiphila rectangulata*, ilPasRect1.1.

Project accession data		
Assembly identifier	ilPasRect1.1	
Species	*Pasiphila rectangulata*	
Specimen	ilPasRect1	
NCBI taxonomy ID	572874	
BioProject	PRJEB63440	
BioSample ID	SAMEA112226468	
Isolate information	ilPasRect1: whole organism (DNA and Hi-C sequencing)	
Assembly metrics *		*Benchmark*
Consensus quality (QV)	66.2	*≥ 50*
*k*-mer completeness	100.0%	*≥ 95%*
BUSCO **	C:98.2%[S:97.7%,D:0.5%], F:0.5%,M:1.3%,n:5,286	*C ≥ 95%*
Percentage of assembly mapped to chromosomes	99.78%	*≥ 95%*
Sex chromosomes	Z	*localised * *homologous pairs*
Organelles	Mitochondrial genome: 15.74 kb	*complete single * *alleles*
Raw data accessions		
PacificBiosciences SEQUEL II	ERR11593805	
Hi-C Illumina	ERR11606320	
Genome assembly		
Assembly accession	GCA_963082625.1	
*Accession of alternate * *haplotype*	GCA_963082775.1	
Span (Mb)	582.5	
Number of contigs	107	
Contig N50 length (Mb)	12.6	
Number of scaffolds	51	
Scaffold N50 length (Mb)	20.9	
Longest scaffold (Mb)	33.74	
Genome annotation		
Number of protein-coding genes	17,153	
Number of gene transcripts	17,368	

* Assembly metric benchmarks are adapted from column VGP-2020 of “Table 1: Proposed standards and metrics for defining genome assembly quality” from Rhie *et al.* (2021). ** BUSCO scores based on the lepidoptera_odb10 BUSCO set using version 5.3.2. C = complete [S = single copy, D = duplicated], F = fragmented, M = missing, n = number of orthologues in comparison. A full set of BUSCO scores is available at https://blobtoolkit.genomehubs.org/view/CAUJBF01.1/dataset/CAUJBF01.1/busco.

**Table 2.** Chromosomal pseudomolecules in the genome assembly of *Pasiphila rectangulata*, ilPasRect1.

INSDC accession	Chromosome	Length (Mb)	GC%
OY720235.1	1	33.74	38.5
OY720237.1	2	24.61	38.5
OY720238.1	3	24.08	38.5
OY720239.1	4	23.95	38.5
OY720240.1	5	23.28	38.0
OY720241.1	6	22.73	38.5
OY720242.1	7	22.62	38.5
OY720243.1	8	22.26	38.5
OY720244.1	9	21.76	38.5
OY720245.1	10	21.57	38.5
OY720246.1	11	21.57	38.5
OY720247.1	12	20.9	38.5
OY720248.1	13	20.59	38.5
OY720249.1	14	20.55	38.5
OY720250.1	15	19.94	38.5
OY720251.1	16	19.59	39.0
OY720252.1	17	19.07	39.0
OY720253.1	18	19.03	39.0
OY720254.1	19	18.59	39.0
OY720255.1	20	18.25	39.0
OY720256.1	21	16.6	39.0
OY720257.1	22	14.58	39.0
OY720258.1	23	14.39	39.0
OY720259.1	24	14.18	39.5
OY720260.1	25	12.61	39.0
OY720261.1	26	12.22	39.0
OY720262.1	27	11.28	40.0
OY720263.1	28	10.02	39.5
OY720264.1	29	9.33	40.5
OY720236.1	Z	27.37	38.5
OY720265.1	MT	0.02	19.5

The estimated Quality Value (QV) of the final assembly is 66.2 with *k*-mer completeness of 100.0%, and the assembly has a BUSCO v5.3.2 completeness of 98.2% (single = 97.7%, duplicated = 0.5%), using the lepidoptera_odb10 reference set ( *n* = 5,286).

Metadata for specimens, barcode results, spectra estimates, sequencing runs, contaminants and pre-curation assembly statistics are given at https://links.tol.sanger.ac.uk/species/572874.

## Genome annotation report

The *Pasiphila rectangulata* genome assembly (GCA_963082625.1) was annotated at the European Bioinformatics Institute (EBI) on Ensembl Rapid Release. The resulting annotation includes 17,368 transcribed mRNAs from 17,153 protein-coding and genes ( Table 1; https://rapid.ensembl.org/Pasiphila_rectangulata_GCA_963082625.1/Info/Index).

## Methods

### Sample acquisition and nucleic acid extraction

A male *Pasiphila rectangulata* (specimen ID Ox002242, ToLID ilPasRect1) was collected from Bratton, Somerset, UK (latitude 51.20, longitude –3.51) on 2022-06-20, using a light trap. The specimen was collected and identified by Denise Wawman (University of Oxford) and then preserved on dry ice.

The workflow for high molecular weight (HMW) DNA extraction at the Wellcome Sanger Institute (WSI) includes a sequence of core procedures: sample preparation; sample homogenisation, DNA extraction, fragmentation, and clean-up. The sample was prepared at the WSI Tree of Life Core Laboratory: the ilPasRect1 sample was weighed and dissected on dry ice ( Jay *et al.*, 2023), and tissue from the whole organism was homogenised using a PowerMasher II tissue disruptor ( Denton *et al.*, 2023a).

HMW DNA was extracted in the WSI Scientific Operations core using the Automated MagAttract v2 protocol ( Oatley *et al.*, 2023). The DNA was sheared into an average fragment size of 12–20 kb in a Megaruptor 3 system ( Bates *et al.*, 2023). Sheared DNA was purified by solid-phase reversible immobilisation ( Strickland *et al.*, 2023): in brief, the method employs a 1.8X ratio of AMPure PB beads to sample to eliminate shorter fragments and concentrate the DNA. The concentration of the sheared and purified DNA was assessed using a Nanodrop spectrophotometer and Qubit Fluorometer and Qubit dsDNA High Sensitivity Assay kit. Fragment size distribution was evaluated by running the sample on the FemtoPulse system.

Protocols developed by the WSI Tree of Life laboratory are publicly available on protocols.io ( Denton *et al.*, 2023b).

### Sequencing

Pacific Biosciences HiFi circular consensus DNA sequencing libraries were constructed according to the manufacturers’ instructions. DNA sequencing was performed by the Scientific Operations core at the WSI on a Pacific Biosciences SEQUEL II instrument. Hi-C data were also generated from remaining tissue of ilPasRect1 using the Arima2 kit and sequenced on the Illumina NovaSeq 6000 instrument.

### Genome assembly, curation and evaluation

Assembly was carried out with Hifiasm ( Cheng *et al.*, 2021) and haplotypic duplication was identified and removed with purge_dups ( Guan *et al.*, 2020). The assembly was then scaffolded with Hi-C data ( Rao *et al.*, 2014) using YaHS ( Zhou *et al.*, 2023). The assembly was checked for contamination and corrected as described previously ( Howe *et al.*, 2021). Manual curation was performed using HiGlass ( Kerpedjiev *et al.*, 2018) and PretextView ( Harry, 2022). The mitochondrial genome was assembled using MitoHiFi ( Uliano-Silva *et al.*, 2023), which runs MitoFinder ( Allio *et al.*, 2020) or MITOS ( Bernt *et al.*, 2013) and uses these annotations to select the final mitochondrial contig and to ensure the general quality of the sequence.

A Hi-C map for the final assembly was produced using bwa-mem2 ( Vasimuddin *et al.*, 2019) in the Cooler file format ( Abdennur & Mirny, 2020). To assess the assembly metrics, the *k*-mer completeness and QV consensus quality values were calculated in Merqury ( Rhie *et al.*, 2020). This work was done using Nextflow ( Di Tommaso *et al.*, 2017) DSL2 pipelines “sanger-tol/readmapping” ( Surana *et al.*, 2023a) and “sanger-tol/genomenote” ( Surana *et al.*, 2023b). The genome was analysed within the BlobToolKit environment ( Challis *et al.*, 2020) and BUSCO scores ( Manni *et al.*, 2021; Simão *et al.*, 2015) were calculated.

Table 3 contains a list of relevant software tool versions and sources.

**Table 3.** Software tools: versions and sources.

Software tool	Version	Source
BlobToolKit	4.2.1	https://github.com/blobtoolkit/blobtoolkit
BUSCO	5.3.2	https://gitlab.com/ezlab/busco
Hifiasm	0.19.5-r587	https://github.com/chhylp123/hifiasm
HiGlass	1.11.6	https://github.com/higlass/higlass
Merqury	MerquryFK	https://github.com/thegenemyers/MERQURY.FK
MitoHiFi	3	https://github.com/marcelauliano/MitoHiFi
PretextView	0.2	https://github.com/wtsi-hpag/PretextView
purge_dups	1.2.5	https://github.com/dfguan/purge_dups
sanger-tol/genomenote	v1.0	https://github.com/sanger-tol/genomenote
sanger-tol/readmapping	1.1.0	https://github.com/sanger-tol/readmapping/tree/1.1.0
YaHS	1.2a.2	https://github.com/c-zhou/yahs

### Genome annotation

The BRAKER2 pipeline ( Brůna *et al.*, 2021) was used in the default protein mode to generate annotation for the *Pasiphila rectangulata* assembly (GCA_963082625.1) in Ensembl Rapid Release at the EBI.

### Wellcome Sanger Institute – Legal and Governance

The materials that have contributed to this genome note have been supplied by a Darwin Tree of Life Partner. The submission of materials by a Darwin Tree of Life Partner is subject to the **‘Darwin Tree of Life Project Sampling Code of Practice’**, which can be found in full on the Darwin Tree of Life website here. By agreeing with and signing up to the Sampling Code of Practice, the Darwin Tree of Life Partner agrees they will meet the legal and ethical requirements and standards set out within this document in respect of all samples acquired for, and supplied to, the Darwin Tree of Life Project. 

Further, the Wellcome Sanger Institute employs a process whereby due diligence is carried out proportionate to the nature of the materials themselves, and the circumstances under which they have been/are to be collected and provided for use. The purpose of this is to address and mitigate any potential legal and/or ethical implications of receipt and use of the materials as part of the research project, and to ensure that in doing so we align with best practice wherever possible. The overarching areas of consideration are:

•   Ethical review of provenance and sourcing of the material

•   Legality of collection, transfer and use (national and international)

Each transfer of samples is further undertaken according to a Research Collaboration Agreement or Material Transfer Agreement entered into by the Darwin Tree of Life Partner, Genome Research Limited (operating as the Wellcome Sanger Institute), and in some circumstances other Darwin Tree of Life collaborators.

## Data Availability

European Nucleotide Archive: *Pasiphila rectangulata*. Accession number PRJEB63440; https://identifiers.org/ena.embl/PRJEB63440 ( Wellcome Sanger Institute, 2023). The genome sequence is released openly for reuse. The *Pasiphila rectangulata* genome sequencing initiative is part of the Darwin Tree of Life (DToL) project. All raw sequence data and the assembly have been deposited in INSDC databases. Raw data and assembly accession identifiers are reported in Table 1.

## Author information

Members of the University of Oxford and Wytham Woods Genome Acquisition Lab are listed here: https://doi.org/10.5281/zenodo.7125292.

Members of the Darwin Tree of Life Barcoding collective are listed here: https://doi.org/10.5281/zenodo.4893703.

Members of the Wellcome Sanger Institute Tree of Life Management, Samples and Laboratory team are listed here: https://doi.org/10.5281/zenodo.10066175.

Members of Wellcome Sanger Institute Scientific Operations: Sequencing Operations are listed here: https://doi.org/10.5281/zenodo.10043364.

Members of the Wellcome Sanger Institute Tree of Life Core Informatics team are listed here: https://doi.org/10.5281/zenodo.10066637.

Members of the Tree of Life Core Informatics collective are listed here: https://doi.org/10.5281/zenodo.5013541.

Members of the Darwin Tree of Life Consortium are listed here: https://doi.org/10.5281/zenodo.4783558.
