# Supplementary material for: The genome sequence of the Green Pug moth, Pasiphila rectangulata (Linnaeus, 1758)
Source: Wellcome Open Res. 2025 Oct 6;9:256. Originally published 2024 May 15. [Version 2] doi: 10.12688/wellcomeopenres.21224.2 (PMC12569505; doi:10.12688/wellcomeopenres.21224.2)
Supplement: Peer review history

# Reviewer report: Reviewer response for version 2

**DOI:** 10.21956/wellcomeopenres.27603.r135459

**Published:** 2025-10-28

**Authors:** Pritha Dey

**Affiliations:** 1 National Centre for Biological Sciences-TIFR, Bengaluru, India

This manuscript presents a high-quality genome assembly of *Pasiphila rectangulata* (Green Pug), accompanied by scaffolding to near-chromosome level, mitochondrial genome assembly, and Ensembl-based gene annotation.

 The work is technically sound and meets the standards expected for genome reports.

 The manuscript is clearly written, well structured, and provides a valuable genomic resource for Lepidoptera research, especially given the relative paucity of chromosome-level genomes among geometrids. I do not see any major methodological or interpretive issues.

 However, I felt the figures are less intuitive, specially Figure 3. If the respective axes of the plot can be explained better for readers, it would be really helpful.

**Recommendation:** Accept as suitable for indexing in its current form.

Are sufficient details of methods and materials provided to allow replication by others?

Yes

Is the rationale for creating the dataset(s) clearly described?

Yes

Are the datasets clearly presented in a useable and accessible format?

Yes

Are the protocols appropriate and is the work technically sound?

Yes

Reviewer Expertise:

Geometridae moth taxonomy, and evolutionary ecology

I confirm that I have read this submission and believe that I have an appropriate level of expertise to confirm that it is of an acceptable scientific standard.

# Reviewer report: Reviewer response for version 1

**DOI:** 10.21956/wellcomeopenres.23471.r91233

**Published:** 2024-08-29

**Authors:** Ananna Ghosh

**Affiliations:** 1 Scientific officer, Insect Biotechnology Division, Atomic Energy Research Establishment, Dhaka, Dhaka Division, Bangladesh

To make the outcome more palatable, the abstract needs to be a bit more detailed. The genome annotation report and genome sequence are both excellent. A bit more work should go into the procedure section, especially the nanodrop quantification result.

 Overall, the research is  commendable and should be approved for indexing.

Are sufficient details of methods and materials provided to allow replication by others?

Partly

Is the rationale for creating the dataset(s) clearly described?

Yes

Are the datasets clearly presented in a useable and accessible format?

Yes

Are the protocols appropriate and is the work technically sound?

Yes

Reviewer Expertise:

Entomology, Molecular biology and Bioinformatics

I confirm that I have read this submission and believe that I have an appropriate level of expertise to confirm that it is of an acceptable scientific standard.

# Reviewer report: Reviewer response for version 1

**DOI:** 10.21956/wellcomeopenres.23471.r91231

**Published:** 2024-08-25

**Authors:** Arjen Van 't Hof

**Affiliations:** 1 Biology Centre of the Czech Academy of Sciences, Institute of Entomology, České Budějovice, Czech Republic

The GCA_963082625.1 assembly of the Green Pug moth is of high quality like we are used to from the Darwin Tree of Life. The assembly statistics show that everything is in order. The assembly is based on a single male according to the manuscript, but the SAMEA112226468 BioSample details show: sex NOT COLLECTED (https://www.ebi.ac.uk/ena/browser/view/SAMEA112226468) ^[ 1]^. Confusingly, the sequenced male has two different BioSample codes, SAMEA112226468 and SAMEA112232922 with only the first listed in Table 1.

 The identification of the Z chromosome is not explained well and based on a string of assumptions.

 It is impossible to understand the following sentence without background knowledge.

 “Chromosome Z was assigned based on synteny with Perizoma affinitatum (GCA_961405105.1) and Camptogramma bilineatum (GCA_958496255.1).”

 What is missing here is an explanation that there is a large body of evidence that chromosomal gene content is conserved in Lepidoptera and can therefore be used to identify homologous chromosomes based on shared synteny. Some references supporting this would also be welcome. I do not object to the ‘assumption-based’ shared synteny Z-chromosome assignment as long as an ‘evidence-based’ Z chromosome is used for comparison. One might expect that the assembly used for shared synteny-based Z chromosome assignment was based on a female with a Z chromosome identified by having half the sequence read coverage of autosomes.

 But following GCA_961405105 and GCA_958496255 leads to surprising trails, which do not end with evidence-based Z-chromosome assigned assemblies.

**Following the Perizoma affinitatum (GCA_961405105.1) trial:**

 Perizoma affinitatum (GCA_961405105.1):

 Chromosome Z was assigned by synteny to Perizoma flavofasciatum (GCA_958496245.1).

 Perizoma flavofasciatum (GCA_958496245.1):

 Z chromosome identified based on synteny with Eulithis prunata (GCA_918843925.1).

 The Eulithis prunata genome was sequenced from one male. It is not explained how the Z chromosome was identified in (https://doi.org/10.12688/wellcomeopenres.19371.1) Boyes *etal*. (2023 ^2^ ), except for “ *localised homologous pairs”* mentioned in Table 1

 This is where the trail starting with Perizoma affinitatum ends without any satisfactory explanation how the Z chromosome was identified in any of the species.

**Following the Camptogramma bilineatum (GCA_958496255.1) trail:**

 Camptogramma bilineatum (GCA_958496255.1):

 Chromosome Z was assigned by synteny to GCA_951804965.1 (Horisme vitalbata)

 Horisme vitalbata (GCA_951804965.1):

 Z chromosome was assigned based on synteny to GCA_949320045.1 (Scotopteryx bipunctaria)

 Scotopteryx bipunctaria (GCA_949320045.1)

 Z chromosome identified based on synteny with Gandaritis pyraliata (GCA_947859175.1)

 The Gandaritis pyraliata (GCA_947859175.1) assembly paper (https://doi.org/10.12688/wellcomeopenres.19526.1) Boyes *etal*. (2023 ^3^ ) does not explain how the Z chromosome was identified. The Z chromosome is mentioned in the abstract, but besides that, all that can be found on sex chromosomes is in table 1:

 Sex chromosomes, W and X chromosomes, localised homologous pairs

 The presence of an X chromosome in Lepidoptera is obviously incorrect, but this review is not about the Gandaritis pyraliata assembly paper.

 The Z chromosome scaffold for the Green Pug moth is probably correct despite the indirect identification. A direct comparison would have been more convincing though.

Are sufficient details of methods and materials provided to allow replication by others?

Partly

Is the rationale for creating the dataset(s) clearly described?

Yes

Are the datasets clearly presented in a useable and accessible format?

Partly

Are the protocols appropriate and is the work technically sound?

Yes

Reviewer Expertise:

Lepidoptera genetics

I confirm that I have read this submission and believe that I have an appropriate level of expertise to confirm that it is of an acceptable scientific standard, however I have significant reservations, as outlined above.

## References

[1] Biosample: SAMEA112226468. Reference source

[2] : The genome sequence of the Phoenix, Eulithis prunata (Linnaeus, 1758). *Wellcome Open Res* .2023;8: 10.12688/wellcomeopenres.19371.1 185 10.12688/wellcomeopenres.19371.1 38404637 PMC10891429

[3] : The genome sequence of the Barred Straw, Gandaritis pyraliata (Denis & Schiffermüller, 1775). *Wellcome Open Research* .2023;8: 10.12688/wellcomeopenres.19526.1 10.12688/wellcomeopenres.19526.1 PMC1281172541550274

# Response

**Published:** 2025-09-28

**Authors:** Tree of Life Team Sanger

**Affiliations:** Wellcome Trust Sanger Institute, UK

Thank you for your careful review of this data note. We have submitted a new version of the article and our responses to your comments are given below.

1. The assembly is based on a single male according to the manuscript, but the SAMEA112226468 BioSample details show: sex NOT COLLECTED (https://www.ebi.ac.uk/ena/browser/view/SAMEA112226468). Confusingly, the sequenced male has two different BioSample codes, SAMEA112226468 and SAMEA112232922 with only the first listed in Table 1.

*Response: This is a standard aspect of the structure of data at the European Nucleotide Archive BioSamples Database. One of the BioSample accessions (SAMEA112226468) identifies the individual or specimen, while the other (SAMEA112232922) identifies the part of the specimen that was used. This is useful if the specimen is dissected into multiple parts, as it can identify different parts used for different sequencing technologies. In this case, the whole organism was used. *  

1. The identification of the Z chromosome is not explained well and based on a string of assumptions. What is missing here is an explanation that there is a large body of evidence that chromosomal gene content is conserved in Lepidoptera and can therefore be used to identify homologous chromosomes based on shared synteny. Some references supporting this would also be welcome. I do not object to the ‘assumption-based’ shared synteny Z-chromosome assignment as long as an ‘evidence-based’ Z chromosome is used for comparison. One might expect that the assembly used for shared synteny-based Z chromosome assignment was based on a female with a Z chromosome identified by having half the sequence read coverage of autosomes.

 
*Response: We identify Z-linked scaffolds by locating BUSCO genes known to be Z-linked across specimens, including heterogametic (ZW) individuals where the Z is unambiguous. In version 2 of the data note we have also applied Merian painting, which shows conserved Z synteny: ancestral Z-linked orthologues co-locate on this chromosome. We have provided a brief explanation and a reference for this paper in the text.*  

1. It is impossible to understand the following sentence without background knowledge.
  “Chromosome Z was assigned based on synteny with Perizoma affinitatum (GCA_961405105.1) and Camptogramma bilineatum (GCA_958496255.1).”

*Response: We have rephrased this slightly in Version 2.*
